# Stigma Among Nurses Toward Individuals with Mental Health Conditions: An Integrative Review of Qualitative and Quantitative Studies

**DOI:** 10.3390/nursrep16020050

**Published:** 2026-01-31

**Authors:** Ruth-Auxiliadora Díaz-Melián, Jesús-Manuel Quintero-Febles, Alfonso-Miguel García-Hernández

**Affiliations:** 1 University Hospital of the Canary Islands, 38320 San Cristóbal de La Laguna, Spain; rdiamelx@gobiernodecanarias.org; 2 Faculty of Nursing, University of La Laguna, 38200 San Cristóbal de La Laguna, Spain; almigar@ull.edu.es

**Keywords:** social stigma, mental disorders, nurses, health personnel, attitude of health personnel

## Abstract

**Background:** Individuals with mental health conditions frequently experience stigmatization and discrimination. Among the primary objectives in the fight against stigma is to examine groups that play a crucial role in addressing it, such as healthcare professionals. Although research has examined stigma among healthcare professionals, few studies have specifically addressed how nurses perceive and contribute to the stigmatization of individuals with mental health conditions. **Objective:** The aim of this review was to compile and compare the scientific literature addressing nurses’ stigma toward individuals with mental health conditions. **Methods:** Following the methodological guidelines of the Joanna Briggs Institute and in accordance with the PRISMA 2020 guidelines, an integrative review was conducted of MEDLINE (PubMed), EMBASE, APA PsycInfo (EBSCO), and CINAHL Complete (EBSCO). Database-specific indexing terms were combined with the Boolean operators AND/OR. Studies with quantitative or qualitative methodologies, published in Spanish or English and without restrictions by year of publication, were included. Two independent reviewers selected the studies and performed the critical appraisal. **Results:** The search retrieved 4256 records, of which 32 articles were finally included. A content analysis of the selected studies was conducted. Most studies used validated questionnaires to assess stigma and its associations with various variables, while only a limited number employed qualitative designs. Across the 32 studies (n = 6283 nurses from 29 countries), stigma was observed across settings but tended to be lower among mental health specialists. Insufficient training and limited contact were consistently associated with higher levels of stigma, whereas specialization and positive contact were linked to lower levels. Associative stigma emerged as a recurrent theme with implications for psychiatric nursing identity. **Conclusions:** Nurses working in mental health settings generally demonstrate more positive attitudes toward individuals with mental health conditions compared with those in other clinical areas; however, stigma persists across all settings. Associative stigma may be influencing the development and advancement of psychiatric nursing. Specific academic training, capacity building, and specialization in mental health are essential to counteract stigma. Further qualitative research is required to achieve a deeper understanding of this phenomenon.

## 1. Introduction

Mental health care for individuals and the communities to which they belong is an essential condition for improving quality of life and exercising full citizenship, where rights and responsibilities coexist. As stated in the Helsinki Declaration of the WHO Ministerial Conference on Mental Health in 2005, “there is no health without mental health” [1]. Among the priority areas of care for people with mental health conditions, as recognized in public health action plans, is the fight against stigma [2]. Although it is widely acknowledged that no one is exempt from experiencing some type of mental health conditions during their lifetime, misinformation and lack of awareness about mental health often result in discrimination, rejection, or stigmatization of those affected [3].

Goffman [4] defined stigma as “an attribute that is deeply discrediting,” degrading the person who carries it. Stigmatized individuals are subject to antipathy, discrimination, dehumanization, and harm from others; their social value is diminished due to attributed characteristics that make them supposedly inferior. Stigma therefore renders a person “different” with negative connotations, generating social discredit and disadvantage. It is a complex psychosocial process, shaped by the interaction between two groups: the stigmatizers and the stigmatized. Those who stereotype make attributions toward others, and those who receive this “virtual identity” respond accordingly [5].

Stigma and discrimination discourage people from acknowledging their mental health conditions and from initiating a recovery process. The persistence of myths, prejudices, and stereotypes contributes to the consolidation of stigma and normalizes discriminatory behaviors. When the person’s diversity is not considered, severe violations of rights may occur. Mental health conditions, through the effect of stigma, become a kind of “label” that overshadows the individual, preventing comprehensive recognition and adequate care for specific needs. Thus, having—or being presumed to have—a mental health condition remains a commonly accepted justification for depriving individuals of their rights [3].

The Lancet Commission on Ending Stigma and Discrimination in Mental Health [6] has proposed a definition that includes the three classical levels of stigma (structural, social, and internalized), while also incorporating associative stigma, which was first noted by Goffman [4] but is less frequently studied. Structural stigma refers to laws, policies, and social norms that, when implemented by public or private institutions, restrict the rights and opportunities of individuals. Social stigma occurs when the general population adopts stereotypes about stigmatized groups and acts accordingly. Internalized stigma, or self-stigma, emerges when individuals internalize the prejudices and stereotypes directed toward them and apply these to themselves. Associative stigma refers to the stigma experienced by the relatives and immediate social environment of the stigmatized individual. Among those within this close circuit is the mental health nurse, who plays a key role in supporting patients throughout their recovery process. Mental health professionals themselves also face subtle or overt devaluation from other healthcare sectors and broader society. Furthermore, limited awareness of the effectiveness of available treatments and mistrust toward mental health professionals are incorporated into prevailing social attitudes [7].

Recognizing the harmful consequences of stigmatization, public health authorities have prioritized its eradication in international, national, and community-level strategies. This concern is reflected in the 2023 State of Health in the European Union Report, which adopts a global approach to mental health that includes twenty initiatives, among them the fight against stigma and discrimination [8]. Similarly, the WHO European Framework for Action on Mental Health (EFAMH) 2021–2025 defines itself as a tool that “provides a coherent basis to intensify efforts aimed at integrating, promoting, and protecting mental well-being as a fundamental element in the response to and recovery from COVID-19; combating stigma and discrimination associated with mental health conditions; and advocating for and promoting investment in accessible and quality mental health services” [9].

Stigma acts through multiple psychological and social mechanisms. Attribution theory [10] suggests that healthcare professionals, like the general population, make causal attributions about mental illness (e.g., “patients are dangerous due to inherent character defects” versus “due to neurobiology”). These attributions activate stereotypes influencing behavior [11]. In healthcare organizations, stigmatizing beliefs are reinforced by informal peer communication, institutional policies separating mental health services, and limited contact preventing stereotype disconfirmation [12].

Although previous evidence suggests mental health nurses show more positive attitudes than colleagues in other specialties, stigma toward people with mental disorders remains a significant concern in mental health nursing for several reasons. First, comparative reduction does not equal elimination: even mental health nurses exhibit measurable stigma, though at lower levels. Second, associative stigma operates uniquely: mental health nurses become stigmatization targets within broader healthcare systems [4,6], paradoxically reinforcing subtle stigmatizing behaviors toward patients as defensive responses. Third, care quality implications require not just stigma reduction, but near-complete elimination to ensure dignified, recovery-oriented care.

Among the measures proposed in these strategies is the need to intervene in professional groups responsible for decision-making and direct care of individuals with mental health conditions. The EFAMH specifically emphasizes the importance of raising awareness of stigma among healthcare professionals. Likewise, Spain’s Mental Health Strategy 2022–2026 [3] highlights stigma reduction as a key objective and establishes a comprehensive plan to address stereotypes, prejudices, and discrimination in multiple domains, including healthcare services and professionals.

Within these professional groups, nurses play a fundamental role in caring for individuals with mental health conditions. Their contribution goes beyond providing clinical care, as they also establish a therapeutic relationship essential for recovery. Their responsibilities include symptom assessment, treatment administration, and monitoring of mental health status, thereby contributing to early detection of crises and relapse prevention. Additionally, through health education and emotional support, they promote treatment adherence and assist patients in developing coping strategies. Their holistic approach, which considers the biological, emotional, and social dimensions of illness, makes them key members of interdisciplinary mental health teams.

Although healthcare professionals are expected to provide care based on empathy and understanding, several studies suggest that they, like the general population, may hold certain stigmatizing beliefs and stereotypes toward people with mental health conditions [5]. Such attitudes can subtly influence clinical interactions and the overall quality of mental health care. Therefore, in alignment with the objectives of combating stigmatization, it is necessary to examine the attitudes and beliefs of nurses toward people with mental health conditions. Yet, despite extensive research on healthcare professionals’ stigma, synthesis specific to nurses remains fragmented and incomplete. Prior reviews aggregate mixed professional samples without isolating nursing-specific data, obscuring critical variations across care contexts (acute versus specialized settings) and their implications for psychiatric nursing’s professional identity and advancement. This review addresses these gaps. The present literature review was designed to synthesize the available scientific evidence regarding stigma toward individuals with mental health conditions by the nurses who care for them. The guiding research question is this: Do nurses stigmatize individuals with mental health conditions?

## 2. Materials and Methods

This integrative review was conducted following the methodological guidelines of the Joanna Briggs Institute (JBI) [13], as well as the PRISMA 2020 (Preferred Reporting Items for Systematic Reviews and Meta-Analyses) statement [14] for reporting results. The complete review protocol was registered on OSF (https://osf.io/9mnjc) (accessed on 24 November 2025) [15].

### 2.1. Inclusion and Exclusion Criteria

Based on the JBI guidelines, the PCC strategy (Population, Concept, and Context) was used to define the problem:

- Population: Nurses providing care to individuals with mental health conditions.
- Concept: Stigma toward individuals with mental health conditions.
- Context: Nurses caring for individuals with mental health conditions in their professional practice.

Inclusion criteria: Studies addressing the stigma of nurses toward individuals with mental health conditions were considered, regardless of whether they employed quantitative, qualitative, or mixed-method designs. Articles published in Spanish or English were included, covering all years available up to December 2025.

Exclusion criteria: Studies focusing on other populations—such as students, families, or professionals other than nurses (e.g., physicians, pharmacists, or social workers)—were excluded. We also excluded studies on stigma among mixed populations of healthcare professionals where nurses were included but results were not reported separately. Review articles, editorials, and letters to the editor were also excluded.

### 2.2. Search Strategy and Study Selection

Following JBI recommendations, an initial exploratory search was conducted in MEDLINE (PubMed) and CINAHL (EBSCO), which identified studies meeting the previously described inclusion criteria. Keywords extracted from titles, abstracts, and indexing terms (MeSH—Medical Subject Headings—and CINAHL Headings) were used to develop search strategies for the selected databases.

Comprehensive searches were then conducted in MEDLINE (PubMed), APA PsycInfo (EBSCO), CINAHL Complete (EBSCO), and EMBASE. The literature search was conducted in December 2023 and updated in January 2026. Controlled vocabulary, thesaurus terms, subject headings, and descriptors from each database were combined with free-text terms using the Boolean operators AND and OR. The search strategy was adapted for each database to account for differences in indexing terms, controlled vocabularies, and search syntax. The complete search strategy employed in each database is presented in Table 1 and described in detail in the registered protocol (https://osf.io/9mnjc (accessed on 24 November 2025)).

All retrieved studies were imported into the Rayyan application to facilitate reference management and automatic duplicate removal. Screening and study selection were performed manually. The initial screening of titles and abstracts was conducted independently by two reviewers (RADM, JMQF) Articles that passed this first screening were retrieved in full text for independent assessment by the same reviewers. Any disagreements regarding study inclusion were resolved through consultation with a third reviewer (AMGH).

### 2.3. Quality Appraisal and Data Synthesis

The methodological quality of the included studies was appraised using the Joanna Briggs Institute (JBI) critical appraisal tools. The checklist applied depended on the design of each study: the JBI tool for analytical cross-sectional studies was used for quantitative cross-sectional designs, the JBI checklist for qualitative research was used for qualitative studies, and the JBI checklist for quasi-experimental studies was applied to the quasi-experimental design, the mixed-methods article was appraised using both the qualitative and the cross-sectional checklists.

Two reviewers independently assessed each study, recording item-level judgements as “yes”, “no”, “unclear”, or “not applicable”. Discrepancies were resolved through discussion, and a third reviewer was consulted when consensus could not be reached. An overall quality rating (high, moderate, or low) was assigned to each study based on the number and relevance of methodological limitations identified. The complete JBI appraisal tables for each study are provided as Supplementary Materials.

Given the diversity of study designs, instruments, and outcomes, the findings were synthesized narratively. Quantitative data were organized into structured tables summarizing study characteristics (e.g., setting, sample, instruments used, and findings related to stigma). Qualitative findings were analyzed using content analysis, identifying meaning units, codes, and broader categories related to nurses’ perceptions and experiences of stigma. To enhance the consistency and credibility of the qualitative synthesis, data extraction and interpretation were conducted independently by two reviewers, with iterative comparison of emerging codes and categories across studies. Discrepancies were resolved through discussion and consensus, in line with JBI methodological guidance. The synthesis integrates results across methodologies to provide a comprehensive description of how stigma toward individuals with mental health conditions is manifested and described in nursing contexts. This review follows the PRISMA 2020 reporting guidelines, and the completed PRISMA 2020 checklist is provided as Supplementary Materials.

### 2.4. Assignment of Overall Quality Ratings

After completing item-level assessments using the appropriate JBI checklists, each study was assigned an overall quality rating using the following criteria:

- High quality: ≥75% of applicable criteria met, with no critical methodological flaws (e.g., appropriate design, adequate sampling, clear reporting);
- Moderate quality: 50–74% of criteria met, with some methodological limitations (e.g., incomplete reporting, moderate risk of bias);
- Low quality: <50% of criteria met, with significant methodological concerns that substantially limit interpretability (e.g., no participant description, no quality measures, very small samples without justification).

Provided as Supplementary Materials.

## 3. Results

### 3.1. General Characteristics of the Included Studies

**Figure 1:**
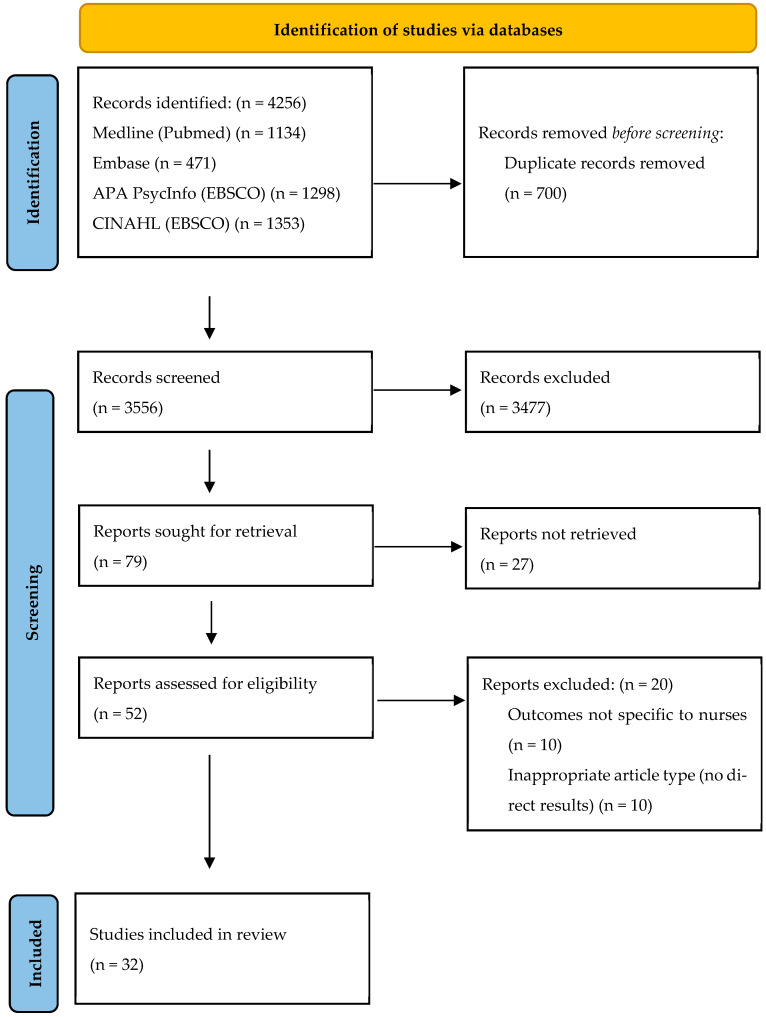
Article selection process following the PRISMA 2020 flow diagram.

The initial search retrieved 4256 records. After removing 700 duplicates, 3556 records were screened by title and abstract. In this first stage, 3477 records were excluded for not meeting the inclusion criteria and 79 studies were selected for full-text review. 27 studies could not be retrieved due to the unavailability of full-text access (restricted access or non-responsive document delivery services), and fifty-two were assessed in full text by two independent reviewers. Of these, 20 studies were excluded for not meeting the inclusion criteria, leaving 32 articles for content analysis. Figure 1 presents a flow diagram, adapted to PRISMA 2020 guidelines, detailing the study selection process.

Data extracted from the 32 studies finally included in the review comprised year of publication, title, authors, research design, study population, relevant findings, and conclusions (Table 2).

Glossary of stigma measurement tools identified across studies:

Caring Behaviors Inventory-24 (CBI-24), Clinicians’ Attitudes Scale, version 4 (MICA v4), Mental Illness Clinicians’ Attitudes Scale (MICA v4.34), Attribution Questionnaire (AQ-9), Caring Behaviors Inventory (CBI-24), Individualized Care Scale for Nurses (ICS-Nurse), Community Attitudes Toward the Mentally Ill (CAMI), Attribution Questionnaire (AQ-27), Psychiatric Patient Perception Scale or Perceived Psychiatric Stigma Scale (PPPS), Depression Anxiety Stress Scales-21 (DASS-21), Attitudes Toward Acute Mental Health Scale (ATAMHS), Community Attitudes Toward the Mentally Ill–Short Form (CAMI-S), Level of Contact (LOC), WHO Health Care Climate for Mental Health (WHO-HC-15-M), National Institute of Mental Health and Neuro Sciences Stigma Index (NIMHANS), Mental Health Problems Perception Questionnaire (MHPPQ), Five Facet Mindfulness Questionnaire (FFMQ), Santa Clara Brief Compassion Scale (SCBCS), Mental Health Attitudes and Knowledge Scale (MAKS), Social Adaptation and Self-Evaluation of Mental Illness Nursing scale (SASMIN), Organizational Mental Health Stigma (OMS-H)

The included studies were published between 2008 and 2025 and conducted across 29 countries: the United States [16,17,18,19], Sweden [20], Jordan [21], Finland [22,23], Lithuania [22], Ireland [22], Italy [22], Portugal [22], Belgium [24,25], Israel [26], Rwanda [27], Kuwait [28], Malaysia [29], Iran [30], Australia [31,32,33], Kenya [34], Ethiopia [35], Brunei [36], India [37], Canada [25,38], France [25], Spain [39,40], Sri Lanka [41], Saudi Arabia [42], Botswana [43], China [44], Netherlands [45], Ghana [46], and Egypt [47].

Most studies employed a quantitative approach [16,18,19,20,21,22,23,25,26,27,28,29,30,33,35,36,37,39,41,43,44,47], whereas fewer used qualitative designs [17,24,31,32,34,40,42,45,46]. One study applied a mixed-methods approach [38].

### 3.2. Quality Appraisal of Included Studies

The methodological quality of the 32 studies included was heterogeneous. The full critical appraisal for each study is provided in the Supplementary Materials.

Among the 22 quantitative studies (21 analytical cross-sectional and one quasi-experimental), several demonstrated high methodological quality, including [18,19,22,33,37,39,43,47], which generally showed clear designs, appropriate procedures and consistent reporting. A larger group of studies [16,20,21,23,25,26,27,28,36,41,44] showed moderate quality, reflecting limitations in design clarity or reporting detail. Two cross-sectional studies, [30,35], were assessed as low quality because of very limited methodological description.

The single quasi-experimental study [29] was judged to be of moderate quality, with an acceptable structure but some methodological limitations that require cautious interpretation.

The qualitative studies were generally stronger: [24,32,40,45] were rated as high quality, while [17,31,34,42,46] showed moderate quality, mainly due to insufficient reporting on reflexivity and researcher positioning.

Finally, the mixed-methods study [38] demonstrated moderate quality in its qualitative component but low quality in its quantitative strand due to limited methodological detail.

### 3.3. Quantitative Studies

Twenty-two quantitative studies [16,18,19,20,21,22,23,25,26,27,28,29,30,33,35,36,37,39,41,43,44,47] were conducted in primary care, emergency services, general hospitals, and specialized mental health settings. The overall sample included 6283 nurses, ranging from studies with 40 participants [33] to multicenter investigations with more than 800 nurses [18,19,22]. Methodologies consisted mainly of cross-sectional designs, multivariate analyses, and regression models to identify significant associations.

The variables related to stigma that were assessed included nursing specialty [16]; familiarity [20]; attitudes and attributions toward mental illness [18,19,21,22,23,26,27,28,29,30,33,35,36,37,39,41]; satisfaction with care provision [21]; contact [25,27]; therapeutic optimism [33]; empathy [33,39,47]; suicide [33]; public stigma [25]; dehumanization [25]; anxiety and depression [25]; burnout [25,39]; caring behaviors [18,19]; individualized care [19]; mindfulness [41]; compassion [41]; social distance [44]; mental health knowledge [44]; and demographic variables (age, sex, academic level).

A total of 29 instruments were used to assess stigma and related variables. The most frequently used instrument was the Community Attitudes Toward the Mentally Ill (CAMI) scale, applied ten times [22,23,26,27,28,30,33,36,37,39], followed by the Mental Illness Clinicians’ Attitudes (MICA-4) scale, used three times [18,19,35,43], and the Attribution Questionnaire (AQ-9), used twice [18,41].

Negative attitudes toward individuals with mental disorders found among nurses [16,20,21,22,26,27,28,30,35] were influenced by perceptions of unpredictability and dangerousness and often associated with factors such as the patients’ expected drug use, alcohol dependence, and criminality [16,21].

Several studies reported that older age and greater professional experience were associated with more positive attitudes toward individuals with mental disorders [16,18,19,20,21,23,24,27,35,39,43], as well as higher levels of empathy [33,39,47], also related to the decrease in the social distance [44]. Gender differences were also observed: male nurses were more likely to display authoritarian attitudes [36], whereas female nurses reported more positive and understanding attitudes [37,39]. Positive attitudes were additionally associated with being female and holding a managerial position [22].

Some studies found that contact with people with mental health conditions reduced stigma and dehumanization, although further research is needed [25]. Importantly, not only direct contact was shown to have a positive effect [30]. A brief contact-based intervention through video projection produced a significant improvement in attitudes in 30% of participants [29]. Familiarity was also identified as a mediating factor in shaping attitudes [27].

Compassion was inversely correlated with avoidance and anger, and positively correlated with pity, helping behaviors, and coercion domains [41]. Trait mindfulness showed a positive correlation with compassion; nurses with higher levels of mindfulness were more likely to believe that they would help a person with mental disorders [41].

A multicenter European study compared attitudes among mental health nurses in five countries and found notable sociocultural differences. While nurses in Portugal reported predominantly positive attitudes, restrictive and stereotypical views persisted in countries such as Lithuania, even among specialized personnel [22].

Specific academic training, professional development, and specialization in mental health emerged as the most effective strategies for counteracting stigmatization [16,18,21,23,26,28,29,33,37,43,44,47].

### 3.4. Qualitative Studies and Mixed-Methods Study

A total of nine qualitative studies and one mixed-methods study, published between 2012 and 2025, were reviewed and analyzed [3,17,24,31,32,34,40,42,45,46], focusing on stigma in medical, psychiatric, emergency, and primary care. The mixed-methods study was incorporated into this synthesis based on the qualitative findings reported. The methods employed included semi-structured interviews, participant observation, and focus groups.

The care of patients seeking help for mental health conditions often generated rejection and fear among nurses, and social stigma was also evident within healthcare environments [40,46]. Nurses expressed frustration at not knowing how to help, although they also showed empathy. They recognized the lack of adequate spaces to individualize treatment, the need for support from specialist nurses, and the importance of continuing education for the management of patients with mental disorders in emergency settings [40].

The negative experiences of medical–surgical nurses in caring for patients with mental health conditions were characterized by tension, discomfort, lack of professional satisfaction, and difficulties in providing care [17]. They recognized limitations in attention and needed to develop skills in the care of patients with mental disorders [45]. Other medical–surgical nurses reinforced stigmatization in hospital environments by categorizing patients with mental health conditions as unpredictable, emotional, or dangerous [32].

The shortage of mental health specialists and limited access to care led nurses to emphasize the need for task-shifting, assuming greater responsibilities in primary care to address the population’s mental health needs. Such measures would optimize available resources, improve overall health, reduce physical deterioration, and provide protection against stigmatization [34].

Studies [24,31] described how mental health nursing is affected by stigma related both to its professional role and to the care model within which it operates. These studies identified several barriers, including institutional policies, unclear work roles, low self-confidence, inadequate support, stress, and insecurity [42,46]. Other findings highlighted concerns regarding limited professional recognition and specialty status, insufficient training and clinical experience during undergraduate education, and limited opportunities for career development [31]. Another finding from the same study [42] was that improving the quality of care was perceived as linked to greater mental health awareness, enhanced skills, and access to continuing education opportunities.

Associative stigma was interwoven throughout the narratives of focus group participants, who highlighted three recurring themes: the perception that mental health nurses are not considered “real” nurses; the lack of recognition of specialized training; and the experience of working with a stigmatized population [38]. The studies described these themes as central aspects of the associative stigma experienced by mental health nurses [38,46]. Structural stigma is related to government neglect and a shortage of resources that devalue psychiatric nursing and hinder the provision of care [46].

### 3.5. Integrated Cross-Study Patterns

Despite diverse methodologies, three robust patterns emerged across the 32 studies:

Setting-dependent stigma gradients: Twelve quantitative studies [18,19,21,23,26,28,30,34,36,44,47] consistently demonstrated more negative attitudes in acute/emergency settings (CAMI >3.0/5) versus psychiatric specialties.

Training deficits as primary drivers: Ten studies [21,23,26,28,30,33,35,39,41,44] confirmed strong associations between lack of mental health training and stigmatizing attitudes (r = 0.25–0.42), most pronounced among less experienced nurses.

Associative stigma’s professional toll: Six studies [16,17,20,24,31,32] evidenced systematic devaluation of psychiatric nursing identity through association with stigmatized patients, rendering it the least preferred specialty.

Key contextual variation: Attitudes deteriorated markedly in low- vs. high-income settings [27,34,35,37,46].

### 3.6. Methodological Heterogeneity and Implications for Interpretation

The 32 included studies varied substantially in design, sample characteristics, instruments, and reported outcomes, which limit direct comparison:

- Design diversity: 21 cross-sectional (mostly descriptive), 1 quasi- experimental, 9 qualitative and one mixed-methods study.
- Instruments: 29 different measurement approaches (10 used CAMI; others unique)
- Sample size: 40–813 participants (median 150).
- Geographic variation: 29 countries across six continents.
- Conceptualization of stigma: Some studies measured explicit attitudes; others measured behavior, contact, or identity issues.
- Outcome definitions: “Stigma” ranged from CAMI scores to behavioral observations to narrative descriptions.

Implications: this heterogeneity means that findings cannot be statistically combined (meta-analysis not appropriate). Instead, we synthesized findings narratively, identifying themes that emerged consistently despite methodological variation. Where quantitative studies used standardized instruments (especially CAMI), we report comparative patterns. Where qualitative studies predominate, we emphasize conceptual insights. Readers should interpret findings as patterns rather than definitive conclusions.

## 4. Discussion

The evidence synthesized in this review of studies examining stigma in nursing highlights that nurses do stigmatize individuals with mental health conditions. Although stigmatization is present across all healthcare settings, fewer stigmatizing attitudes were observed among nurses working in specialized mental health services. Professional experience, specific academic training, and specialization in mental health emerge as the most important measures to reduce stigma.

Associative stigma is also present among mental health nurses and may influence the development and advancement of the discipline. Revaluing the role of mental health nursing within healthcare systems is therefore essential.

Most of the studies included employed quantitative designs, frequently using validated questionnaires to assess stigma, with the Community Attitudes Toward the Mentally Ill (CAMI) being the most common.

The overall interpretation of findings should consider the variability in methodological quality across studies. The qualitative evidence was generally the strongest: high-quality studies [24,32,40] provided consistent, credible insights into nurses’ experiences of stigma, and the moderate-quality studies [17,31,34,42] reinforced these themes despite some reporting limitations.

The quantitative evidence was more heterogeneous. Although several studies demonstrated high quality [18,19,22,33,37,39,43,47], many showed moderate quality [16,20,21,23,26,27,28,36,41,44], and two were rated as low quality [30,35]. These variations limit the strength of inferences that can be drawn from quantitative findings. The quasi-experimental study [29] and the mixed-methods study [38] contributed additional perspectives but required cautious interpretation due to moderate or low methodological rigor.

Taken together, the overall body of evidence can be considered moderate in quality: qualitative studies provide strong conceptual support for the presence and nature of stigma, while quantitative findings are informative but less methodologically consistent.

Mental health nursing is defined by a practice centered on empathy, the understanding of psychological suffering, and therapeutic accompaniment [24]. However, the studies reviewed reveal that stigma toward individuals with mental health conditions may still be present even among those working specifically in this field [21,30,47], challenging the assumption that knowledge and experience automatically ensure a prejudice-free attitude. These findings are consistent with other research examining healthcare professionals’ attitudes toward individuals with mental health conditions [48,49,50]. Evidence shows that even professionals working in mental health [48,49,50,51,52,53,54], despite having greater knowledge, sometimes prefer measures of social distance [52,55,56], suggesting that professional contact alone is insufficient to guarantee the eradication of stigma.

Nevertheless, some studies [26,39,47] found that mental health nurses tend to hold more positive attitudes toward individuals with mental health conditions than their colleagues in other specialties, although this difference is neither absolute nor consistent. Importantly, empathy does not always protect against burnout, which suggests that emotional involvement may be strained by structural working conditions. Such strain may give rise to distancing behaviors as defensive strategies that, indirectly, reinforce stigma [39]. Despite these nuances, multiple studies found that professionals lacking training and experience were more likely to display stigmatizing attitudes [23,25,26,28,33,35,36,37,43,44]. These results were consistent across different care settings—primary care, emergency services, intensive care, hospitalization, and mental health units—as well as across diverse cultural contexts. Together, these findings suggest that training and experience, while not guaranteeing the absence of stigma, are among the most effective measures to combat the stigmatization of people with mental health conditions.

The importance of specialized training in mental health has also been highlighted in qualitative research exploring the negative feelings and prejudices expressed by emergency nurses, who reflected on how they should approach patients with mental health conditions and recognized the need for support from specialist nurses to provide adequate care [40]. Other proposals, such as the practice of mindfulness, may improve attitudes toward coercion and have a buffering effect against stigma by fostering awareness and nonjudgmental acceptance of experiences in caregiving [41]. However, more research is needed to confirm the protective role of mindfulness against stigma.

According to Goffman, associative stigma occurs when individuals or groups closely linked to a stigmatized person also experience stigma “by courtesy” [6]. This concept suggests that mental health nurses not only witness stigma directed at their patients but also become targets of stigmatization within the healthcare system themselves. They are perceived as less competent and engaged in work considered “less scientific” or “less valuable” compared to other specialties [16,46]. Such stigmatization may contribute to the internalization of negative stereotypes, which can in turn affect nurses’ attitudes toward their own patients, sometimes reinforcing perceptions of dangerousness or social threat [26,27,32].

Social and structural stigma [6,46] are also reflected in the lack of institutional support for caregivers and families, insufficient staffing, inadequate treatment facilities, organizational changes, limited awareness among other healthcare professionals, and the absence of protocols to improve care quality [24,34,40,42].

Mental health nursing must therefore make its role more visible, further develop the profession and the recognition of the specialty, remain motivated to counteract stigma, and strengthen the professional identity of nurses in deinstitutionalized contexts. Psychiatric nursing must challenge associative stigma, often perceived as a discipline caught between autonomous practice and its roots in psychiatry. It should embrace an empathic, holistic, and respectful approach while rejecting reductionist models; only then will it be able to break the cycle of stigma [24,31,42].

A key axis of stigmatization is the undervaluation of relational and emotional competencies, which are fundamental to mental health practice. While technical skills—such as medication administration or device management—are often recognized and prioritized in clinical settings, psychosocial skills such as active listening, empathy, emotional support, crisis management, and the building of therapeutic relationships are frequently dismissed as “soft” or secondary. This dichotomy, identified in several of the studies reviewed, reinforces the perception that psychiatric nursing is less demanding or scientific. These competencies must be redefined as essential and sophisticated, representing the core of a specialized practice that requires complex knowledge of psychopathology, psychopharmacology, ethics, therapeutic communication, and community health [31,38]. Taken together, these findings point to the need to further explore how relational competencies might relate to stigma reduction in mental health nursing

Nursing leadership in providing quality care, ensuring safe, dignified, respectful, inclusive, equitable, and compassionate experiences, and supporting recovery is fundamental to improving patient health outcomes [18,19,22,23]. More effective approaches are particularly needed in hospital settings [17,45]. Consequently, the role of mental health nursing is crucial in transforming stigma and marginalization [34], as emphasized in international and national strategies [3,8,9].

In contrast, one consistent theme across the studies is the strong ethical and human commitment demonstrated by specialized mental health nurses. Despite the obstacles they face, many report a profound sense of vocation, empathy, and professional pride. These narratives, although less frequent within the dominant discourse, represent an opportunity to reframe the profession and construct an alternative narrative that challenges stigma and highlights the value of mental health nursing [24,38]. Qualitative research will be essential for deepening the understanding of nurses’ perspectives and examining and interpreting their attitudes, prejudices, and feelings as part of care practice. Perspectives shape the way we see the world and other people. They are rooted in belief systems that unconsciously influence what we do, what we believe, and who we are, ultimately determining our perception of our place in life. Studying stigma through qualitative research could foster change among professionals by encouraging reflection on their caregiving role. Several authors have also emphasized the need to expand qualitative research on this topic [20,28,37]. Further qualitative research is needed to explore the reasons why stigma persists among mental health professionals, examining concepts such as stigma, stereotyping, labeling, negative attitudes, discrimination, and emotional responses, while also strengthening the credibility of nursing interventions.

### 4.1. Implications for Mental Health Nursing Practice and Research

The findings of this review have several implications for mental health nursing practice. Although nurses working in specialized mental health settings generally display more positive attitudes toward individuals with mental health conditions, stigma continues to be manifested across all clinical environments. This suggests that formal training, clinical experience, and specialization—while protective—are not sufficient to eliminate stigmatizing beliefs. Mental health nursing practice should therefore incorporate structured educational interventions that address stigma explicitly, promote reflective practice, and strengthen competencies related to therapeutic communication, empathy, and emotional management. Given the persistent undervaluation of relational and emotional skills, greater institutional recognition of these competencies is essential to support high-quality, person-centered mental health care.

This review also highlights several implications for future mental health nursing research. The predominance of quantitative cross-sectional designs limits the depth of understanding regarding how stigma develops, how it is experienced by nurses, and how it shapes everyday clinical decision-making. Additional qualitative and mixed-methods research is needed to explore factors that may contribute to the persistence of stigma, including emotional responses, contextual pressures, professional identity tensions, and organizational determinants. There is also a need for studies evaluating the effectiveness of interventions—such as specialized training, reflective practice programs, or mindfulness-based strategies—in reducing stigma among nurses working in diverse settings.

Finally, future research should more rigorously assess the quality of evidence and employ longitudinal or quasi-experimental designs where possible, allowing for the examination of changes over time and the identification of factors that contribute to sustained improvements in attitudes and practice. Strengthening this evidence base will support the development of targeted strategies to reduce stigma and enhance the contribution of mental health nursing to recovery-oriented care.

### 4.2. Limitations of the Review

This integrative review presents several limitations that should be considered when interpreting its findings. First, the search was limited to four major databases (MEDLINE, EMBASE, CINAHL, and APA PsycInfo), which may have resulted in the omission of relevant studies indexed elsewhere or published in journals not included in these platforms. Second, only articles published in English or Spanish were included, introducing a potential language bias. Third, although all reasonable efforts were made to retrieve full texts, twenty-seven studies could not be accessed and were therefore excluded, which may have affected the comprehensiveness of the evidence base.

Fourth, the heterogeneity of study designs, populations, and instruments prevented quantitative synthesis, and the narrative synthesis may be subject to interpretative bias despite the use of structured extraction procedures. Fifth, the inclusion of studies with varying methodological quality may have influenced the overall strength of the conclusions; although critical appraisal was conducted using JBI tools, the evidence ultimately depends on the rigor of the original studies. This review did not include grey literature, conference proceedings, or doctoral dissertations, which may limit the breadth of perspectives represented, particularly for qualitative evidence or emerging research themes.

In addition, several limitations related to the nature of the available evidence should be acknowledged. Most quantitative studies employed cross-sectional designs, which precludes causal inference and limits the ability to determine whether factors such as training or specialization actively reduce stigma or whether nurses with more positive attitudes are more likely to seek such training. The predominance of observational designs, together with the scarcity of intervention studies and the limited number of qualitative investigations, constrains conclusions regarding mechanisms of change and the long-term effectiveness of anti-stigma strategies. Consequently, the associations identified in this review should be interpreted as indicative patterns rather than deterministic relationships.

Furthermore, substantial heterogeneity was observed in the conceptualization and measurement of stigma. Across the included studies, a wide range of instruments was used, reflecting differing theoretical frameworks and operational definitions of stigma. This variability, together with differences in qualitative analytic approaches, limits comparability across studies and precluded quantitative synthesis. As a result, the findings of this review represent aggregated trends rather than pooled estimates, and some observations may be specific to particular instruments or contexts.

The representativeness of study samples also warrants caution. Many studies relied on convenience sampling and were conducted predominantly in high-income countries, particularly in Europe and North America, with relatively few investigations from low- and middle-income settings. Given global disparities in mental health burden, healthcare organization, and professional training, these imbalances may affect the generalizability of the findings and potentially underestimate or mischaracterize stigma in underrepresented regions. In this sense, the conclusions of this review should be interpreted with sensitivity to cultural, organizational, and health-system contexts.

Finally, while several studies consistently reported comparatively more positive attitudes among mental health nurses than among nurses in other specialties, this finding requires nuanced interpretation. Lower levels of stigma do not imply the absence of stigmatizing attitudes, and moderate levels of stigma remain prevalent even within specialized mental health settings. Moreover, evidence of associative stigma toward mental health nurses suggests that stigma operates across multiple levels—individual, professional, and structural. Focusing exclusively on patient-directed stigma without addressing the stigmatization of mental health nursing as a specialty may limit the effectiveness of educational and organizational interventions. These considerations further underscore the need for future longitudinal, qualitative, and intervention-based research capable of capturing the complex and multilevel nature of stigma in nursing practice.

### 4.3. Future Research Needs

Although quantitative evidence demonstrates that stigma persists despite training and specialization, qualitative research is urgently needed to understand why. Specifically, we need in-depth exploration of: How do nurses themselves interpret and experience stigma in their daily practice? What emotional and cognitive barriers prevent attitude change despite training? How does associative stigma specifically shape mental health nurses’ professional identity and clinical decision-making? What organizational and structural factors perpetuate stigma despite individual-level interventions? These questions cannot be adequately answered with binary yes/no response scales; they require interpretive, exploratory methodologies that capture the complexity of nurses’ perspectives and experiences. Additionally, qualitative research should examine not only barriers to positive attitudes but also exemplary of anti-stigma practice: what enables some nurses and teams to successfully counter stigma?

Although specialization and experience reduce stigma among nurses, they do not eliminate it. This finding suggests that focusing solely on mental health nurses is insufficient; attention must also be paid to the associative stigma faced by mental health nurses themselves, which may mediate their attitudes toward patients.

## 5. Conclusions

This integrative review shows that stigma toward individuals with mental health conditions persists across nursing settings, although it is generally less pronounced among nurses working in specialized mental health services. Professional experience, specific academic training, and mental health specialization appear to mitigate stigmatizing attitudes, yet these factors remain insufficient to eliminate them completely.

The available evidence suggests that specific academic training and specialization in mental health are associated with reduced stigmatizing attitudes among nurses; however, these factors do not eliminate stigma. Training should therefore be considered a necessary but insufficient component of comprehensive anti-stigma strategies, which also need to incorporate organizational policy changes, structural interventions addressing resource allocation, and ongoing reflexive and educational practices. Importantly, the mechanisms through which training and specialization may influence attitudes remain insufficiently understood, as does the long-term sustainability of attitude change, underscoring the need for further research.

Associative, social, and structural forms of stigma continue to shape the professional identity of mental health nurses and the value attributed to relational competencies—elements that are essential to practice yet frequently undervalued. Enhancing recognition of the specialty, strengthening training pathways, and promoting relational skills may help counteract stigma within healthcare systems.

This review offers a unique contribution by integrating findings from 32 global studies that identify associative, social, and structural stigma as key barriers to the professional identity of mental health nurses and the valuation of essential relational competencies, which are frequently undervalued.

Given the moderate overall quality of the evidence, future research should prioritize rigorous qualitative and mixed-methods designs to clarify why stigma persists despite training and experience, and to assess interventions aimed at reducing stigma in diverse clinical contexts.

By integrating findings from a wide range of settings and methodological perspectives, this review identifies key professional and organizational factors that influence stigma among nurses and provides a foundation for developing evidence-based strategies to improve care and reduce stigmatization.

Nursing leadership emerges as a key agent to eradicate stigma in mental health through multilevel interventions: ongoing educational programs that challenge stereotypes, promotion of supervised positive contact, and institutional policies that allocate specific resources, recognize specialized competencies, and foster professional identity against associative stigma. Aligned with strategies such as the EFAMH 2021–2025 and Spain’s Mental Health Strategy 2022–2026, managers must drive metrics for attitude evaluation, specialist retention, and interprofessional collaboration to elevate care quality and advance the discipline.

## Figures and Tables

**Table 1:** Search strategy used.

Database	Search Strategy
MEDLINE (PUBMED)	(((((stigma*) OR Attitude of Health Personnel [MeSH Terms]) OR social stigma [MeSH Terms])) AND ((((mental disorders [MeSH Terms]) OR Mentally Ill Persons [MeSH Terms]) OR Mentally Ill Persons) OR mental disorders)) AND ((((nurses [MeSH Terms]) OR nursing [MeSH Terms]) OR nursing*) OR nurses*)
APA PsycInfo (EBSCO)	(DE Mental health stigma OR DE Stigma OR DE Health Personnel Attitudes OR DE Self-Stigma OR Stigma*) AND (DE Nurses OR Nurs*) AND (DE Mental Disorders OR DE “Mental Health (Attitudes Toward)” OR Mental Disorders)
CINAHL Complete (EBSCO)	(MH Stigma OR MH Attitude of Health Personnel OR Stigma OR Attitude of Health Personnel) AND (MH Nurses OR Nurs*) AND (Mental disorders OR Mentally Ill Persons OR Mental illness OR MH mental disorders OR MH Persons with Mental Disorders)
EMBASE	(‘stigma’/mj OR ‘social stigma’/mj OR ‘nurse attitude’/mj) AND ‘mental disease’ AND ‘nurse’

**Table 2:** Key data from the selected studies.

Author (Year)	Title	Design/Instruments	Study Population, Country	Main Findings	Conclusions
Halter MJ. (2008) [16]	Perceived characteristics of psychiatric nurses: stigma by association.	Quantitative study/ Nursing Specialty Area Inventory.	122 nurses, EEUU	Psychiatric nursing is the least preferred field. Psychiatric nurses are perceived as inexperienced, illogical, idle, and disrespectful. Presence of stigmatizing attitudes among healthcare professionals toward individuals with mental illness.	Stigmatizing attitudes among nurses are common and may be reinforced through professional training.
Zolnierek CD, Clingerman EM. (2012) [17]	A medical–surgical nurse’s perceptions of caring for a person with severe mental illness.	Qualitative study/descriptive case study.	1 nurse, EEUU	The nurse’s experience was characterized by categories of tension, discomfort, lack of professional satisfaction, and difficulty.	Understanding of nurses’ care experiences can inform efforts to improve practice environments, provide resources, or develop models of care that support nurses who care for patients with SMI and improve health outcomes for people with SMI.
McIntosh JT. (2023) [18]	Emergency department nurses’ perceptions of caring behaviors toward individuals with mental illness: A secondary analysis.	Quantitative study CBI-24, MICA v4	813 emergency nurses, EEUU	Caring behaviors were positively associated with individualized care, while stigma (MICA v4) showed a negative association. Nurses generally reported moderate to high caring behaviors but persistent stigmatizing attitudes toward mental illness.	Caring behaviors enhance individualized care, but stigma remains a barrier. Training and awareness interventions are needed to reduce stigma and strengthen person-centered care for patients with mental illness.
McIntosh JT. (2023) [19]	Illuminating Emergency Nurses’ Perceptions of Stigma, Attribution, and Caring Behaviors Toward People with Mental Illness Through the Lens of Individualized Care: A Cross-sectional Study.	Quantitative study MICA v4.34, AQ-9, CBI-24, ICS-Nurse.	813 emergency nurses, EEUU	Caring behaviors showed a strong positive association with individualized care. Stigma and attribution had weak inverse associations with individualized care. Demographic factors (sex, marital status, region, training, and access to behavioral health resources) also influenced perceptions of individualized care.	Individualized care in emergency settings is influenced more by caring behaviors than by stigma or attribution. While stigma showed only a weak association, it remains present and can negatively affect perceptions. Interventions should prioritize strengthening caring behaviors while also addressing stigma to improve care for patients with mental illness.
Björkman T, Angelman T, Jönsson M. (2008) [20]	Attitudes towards people with mental illness: a cross-sectional study among nursing staff in psychiatric and somatic care.	Quantitative study/Questionnaire on Level of Familiarity with Mental Illness, CAMI	120 nurses, Sweden	More negative attitudes regarding dangerousness and unpredictability in drug addiction, alcohol dependence, and schizophrenia, particularly among younger staff with less professional experience.	Attitudes toward people with mental disorders among psychiatric nursing staff are, in several respects, similar to those of the general population.
Hamdan-Mansour AM, Wardam LA. (2009) [21]	Attitudes of Jordanian mental health nurses toward mental illness and patients with mental illness.	Quantitative study/Acute Mental Health Attitude Scale; nurses’ satisfaction with the provision of mental health care.	92 mental health nurses, Jordan	60% of mental health nurses perceived patients with mental illness as dangerous, immature, unclean, cold, harmful, and pessimistic. Nurses were dissatisfied with the provision of mental health care. Over 70% reported being proud to be mental health nurses. Age and gender were significant factors influencing attitudes and satisfaction.	Factors such as nurses’ training and workplace affected their satisfaction. Highlights the importance of mental health specialization and the need to support interdisciplinary efforts for strategic planning in mental health reform, as well as undergraduate and postgraduate nursing education.
Chambers M, Guise V, Välimäki M, Botelho MA, Scott A, Staniuliené V et al. (2010) [22]	Nurses’ attitudes to mental illness: a comparison of a sample of nurses from five European countries.	Quantitative study CAMI	810 mental health nurses, Finland, Lithuania, Ireland, Italy, and Portugal.	Nurses’ attitudes were mostly positive. Attitudes differed between countries: Portuguese nurses reported significantly more positive attitudes, whereas Lithuanian nurses reported significantly more negative attitudes. Positive attitudes were associated with being female and holding a managerial position.	Although European mental health nurses’ attitudes toward people with mental disorders differ significantly between some countries, they are largely similar overall. Observed differences may be related to broader social, cultural, and organizational circumstances of nursing practice.
Ihalainen-Tamlander N, Vähäniemi A, Löyttyniemi E, Suominen T, Välimäki M. (2016) [23]	Stigmatizing attitudes in nurses towards people with mental illness: a cross-sectional study in primary settings in Finland.	Quantitative study AQ-27	264 primary care nurses, Finland	The nurses mostly reported willingness to help and feelings of concern and sympathy towards these patients. However, younger nurses or those without additional mental health training expressed a fear of patients.	Special attention should be paid to nursing education and on-the-job training to prevent young nurses from developing stigmatized attitudes towards patients. Implications for practice: Higher confidence in nursing staff could ensure a skilled work force in areas of mental health in the future, prevent young nurses from developing a fear of patients at work and support positive attitudes towards patients with mental problems.
Sercu C, Ayala RA, Bracke P. (2015) [24]	How does stigma influence mental health nursing identities? An ethnographic study of the meaning of stigma for nursing role identities in two Belgian Psychiatric Hospitals.	Qualitative study/Participant observation and semi-structured interviews.	92 mental health nurses, Belgium	Tackling stigma is a particularly important personal motive for nurses to work in mental health care. The meaning of stigma is closely entangled with nurses’ troublesome relationship with the medical model of care. Variations between hospitals regarding the extent to which stigma informs the professional role constructs and identity of nurses are found to be related to the degree of formalization of the nursing roles in these different hospitals.	The study points to the relevance of the integration of stigma in mental health nursing identity research. Furthermore, the focus on stigma may offer an opportunity to link contexts of illness and care, and nurses’ identity constructs.
Fontesse S, Rimez X, Maurage P. (2021) [25]	Stigmatization and dehumanization perceptions towards psychiatric patients among nurses: A path-analysis approach.	Quantitative study/PPPS, Dehumanization Scale, Quality of Contact Scale, Maslach Burnout Inventory, DASS-21; Moral dilemmas; Diagnostic Overshadowing task, Resource Allocation task.	336 nurses, Belgium, France and Canada	Higher perceived stigma was associated with stronger dehumanization of patients and lower quality of contact. Dehumanization correlated with greater burnout, stress, anxiety, and depressive symptoms. Structural stigma was evident through discriminatory resource allocation.	Findings highlight the role of dehumanization and associative stigma in nursing practice. Improving contact quality and addressing burnout are key strategies to reduce stigma and enhance mental health care.
Ben Natan M, Drori T, Hochman O. (2015) [26]	Associative Stigma Related to Psychiatric Nursing Within the Nursing Profession.	Quantitative study/CAMI, ATAMHS	108 psychiatric nurses and 108 non-psychiatric nurses, Israel	Psychiatric nurses showed more positive attitudes, whereas non-psychiatric nurses doubted their ability to provide valuable psychiatric nursing care. Older non-psychiatric nurses exhibited higher levels of stigma toward mental illness.	Stigma toward mental illness was more frequent among non-psychiatric nurses. Associative stigma was present in both groups.
Vedaste B, Smith A A H. (2016) [27]	In principle, yes, in application, no’: Rwandan nurses’ support for integration of mental health services.	Quantitative study/LOC, CAMI-S	102 nurses, Rwanda	Stigmatizing attitudes toward mental illness were present among Rwandan nurses. Familiarity had mediating effects. Significant associations were found between mental illness stereotypes and younger, less experienced nurses. Contradictions in CAMI-S responses across demographic variables suggest a tension between nurses’ professional identity and the ideology of non-discrimination.	Intervention studies are needed to provide empirical data on the effectiveness of introducing narrative approaches and whether such interventions facilitate progress and community integration of mental health services.
Al-Awadhi A, Atawneh F, Alalyan MZY, Shahid AA, Al-Alkhadhari S, Zahid MA. (2017) [28]	Nurses’ attitude towards patients with mental illness in a general hospital in Kuwait.	Quantitative study CAMI.	308 nurses, Kuwait	The mean scores for the subscales reflected a negative attitude of nurses toward mentally ill patients. The direct or indirect utilization of the mental health facilities resulted in significantly higher authoritarian and lower benevolence scores, indicating a positive attitude change in this group of nurses.	The nurses’ negative attitude toward the mentally ill patients provides useful baseline data for further large-scale studies and underscores the need for psychoeducation of different health care professionals, including nurses.
Ng YP, Rashid A, O’Brien F. (2017) [29]	Determining the effectiveness of a video-based contact intervention in improving attitudes of Penang primary care nurses towards people with mental illness.	Quasi-experimental study/WHO-HC-15-M and the VBCI video-based intervention.	206 primary care nurses, Malaysia	Pre–post VBCI score differences were statistically significant, with a 14% reduction. VBCI significantly improved attitudes in 30% of participants. Less stigmatizing baseline attitudes were associated with prior psychiatric training, interest in psychiatric training, and positive contact with people with mental illness.	A brief VBCI is effective in improving attitudes of primary care nurses towards people with mental illness in the immediate term.
Ebrahimi H, Jafarabadi MA, Areshtanab HN, Pourabbas M, Dehghan A, Vahidi M. (2017) [30]	Comparing mental illness stigma among nurses in psychiatric and non-psychiatric wards in Tabriz University of medical sciences.	Quantitative study CAMI.	93 psychiatric ward nurses and 105 non-psychiatric nurses, Iran	No significant differences in stigma scores were observed between the two groups. Contact with people with mental illness was not associated with positive attitudes. Among non-psychiatric ward nurses, 86.7% reported they would not like to work in psychiatric wards in the future, while 63.3% of psychiatric ward nurses expressed willingness to continue working there.	Working in mental health alone does not generate positive attitudes; the quality and quantity of contact are more effective in reducing stigma.
Harrison CA, Hauck Y, Ashby R. (2017) [31]	Breaking down the stigma of mental health nursing: A qualitative study reflecting opinions from Western Australian nurses.	Qualitative study/semi-structured interviews.	192 mental health nurses, Australia	Stigma and low visibility hinder recruitment and retention in mental health nursing. Identified needs include greater promotion, positive student experiences, and professional recognition of the specialty.	Breaking down stigma is essential to strengthen the professional identity and sustainability of mental health nursing through education, institutional support, and recognition as a specialty.
Brunero S, Buus N, West S. (2017) [32]	Categorising Patients Mental Illness by Medical Surgical Nurses in the General Hospital Ward: A Focus Group Study.	Qualitative study/focus groups.	16 nurses, Australia	Four categories of mentally ill patients: the managed, the unpredictable, the emotional and the dangerous.	The language used by medical/surgical reflects the wider discourse of managerialism in healthcare organizations. The recognition of these categories can be used by educators, liaison mental health services and policy makers to reconsider service design and learning opportunities for medical surgical nurses to reduce stigmatization of patients with mental illness.
Weare R, Green C, Olasoji M, Plummer V. (2019) [33]	ICU nurses feel unprepared to care for patients with mental illness: A survey of nurses’ attitudes, knowledge, and skills.	Quantitative study/Therapeutic Optimism Scale, the Jefferson Scale of Physician Empathy, the Attitudes Toward Suicide Questionnaire, CAMI.	40 ICU nurses, Australia	ICU nurses in Melbourne reported generally negative attitudes toward patients with mental illness, often perceiving them as unpredictable or difficult to manage. Empathy and therapeutic optimism were limited, while stigma and skepticism regarding recovery were common.	Specialized training and psychoeducation are needed to improve empathy, reduce stigma, and strengthen ICU nurses’ capacity to provide appropriate mental health care.
Mendenhall E, Isaiah G, Nelson B, Musau A, Koon AD, Smith L et al. (2018) [34]	Nurses’ perceptions of mental healthcare in primary-care settings in Kenya.	Qualitative study/semi-structured interviews.	60 nurses, Kenya	Nurses identified cost, stigma, cultural beliefs, and lack of specialists as key barriers to mental healthcare. Most supported integrating mental health into primary care as acceptable and feasible, with nurses playing a central role in delivery.	Task-sharing mental health services with nurses in primary care is a promising strategy to address Kenya’s treatment gap, though training, supervision, and resources are critical for effective implementation.
Sahile Y, Yitayih S, Yeshanew B, Ayelegne D, Mihiretu A. (2019) [35]	Primary health care nurses’ attitude towards people with severe mental disorders in Addis Ababa, Ethiopia: A cross-sectional study.	Quantitative study/NIMHANS, MICA-4.	610 primary care nurses, Ethiopia	Negative attitudes were reported by 48.2% of nurses; predictors included lower education (diploma), <5 years of experience, no mhGAP training, and poor knowledge of mental illness.	Nearly half of the participants have a negative attitude towards people with severe mental disorders. Therefore, evidence-based and contextualized models are warranted to mitigate negative attitudes of primary health care nurses.
Shahif S, Idris DR, Lupat A, Abdul Rahman H. (2019) [36]	Knowledge and attitude towards mental illness among primary healthcare nurses in Brunei: A cross-sectional study.	Quantitative study/CAMI, MHPPQ	62 primary care nurses, Brunei	Knowledge was positively correlated with authoritarianism and inversely with social restrictiveness, no significant correlation with benevolence. Higher educational level was associated with authoritarian attitudes.	Negative attitudes among nurses remain a challenge; re-education initiatives and increased contact time are needed to foster attitude change and support holistic mental health care.
Grover S, Sharma N, Mehra A. Stigma for Mental Disorders among Nursing Staff in a Tertiary Care Hospital. (2020) [37]	Stigma for Mental Disorders among Nursing Staff in a Tertiary Care Hospital.	Quantitative study CAMI.	210 nurses, India	Overall attitudes toward people with mental illness were generally positive. Female nurses scored higher on social restrictiveness, while no significant associations were found with age, education, or prior experience. Benevolence correlated positively with all CAMI domains.	Nurses showed ambivalent but largely sympathetic attitudes. Findings highlight the need for targeted mental health awareness campaigns and ongoing education to strengthen positive attitudes and reduce stigma.
Waddell C, Graham JM, Pachkowski K, Friesen H. (2020) [38]	Battling Associative Stigma in Psychiatric Nursing.	Mixed-methods/14-item survey, focus groups.	94 psychiatric nurses, Canada	Three themes emerged: psychiatric nurses perceived as not “real” nurses; lack of recognition of their specialized training; and challenges of working with a stigmatized population. Associative stigma was evident across narratives.	Associative stigma undermines psychiatric nurses’ identity and professional recognition. Addressing it requires redefining “soft skills” as essential, promoting the unique contributions of psychiatric nurses, and embedding anti-stigma strategies in education and professional practice.
Román-Sánchez D, Paramio-Cuevas JC, Paloma-Castro O, Palazón-Fernández JL, Lepiani-Díaz I, de la Fuente Rodríguez JM et al. (2022) [39]	Empathy, Burnout, and Attitudes towards Mental Illness among Spanish Mental Health Nurses.	Quantitative study/Jefferson Empathy Scale, Maslach Burnout Inventory, CAMI.	750 mental health nurses, Spain	High empathy among Spanish mental health nurses correlated with benevolence and positive attitudes, but also with greater burnout (emotional exhaustion and depersonalization.	Empathy reduces stigma but does not protect against burnout; interventions are needed to strengthen empathy and mitigate burnout.
García-Carpintero Blas E, Gómez-Moreno C, Moreno-Gomez-Toledano R, Ayuso-Del-Olmo H, Rodrigo-Guijarro E, Polo-Martínez S et al. (2023) [40]	Help! Caring for People with Mental Health Problems in the Emergency Department: A Qualitative Study.	Qualitative study/semi-structured interviews.	15 emergency nurses, Spain	Emergency nurses reported fear, mistrust, and stigma toward patients with mental illness, often leading to rejection or use of restraints. Barriers included overload, lack of time, inadequate spaces, and absence of protocols, worsened during COVID-19. Empathy was more common among nurses with personal or family experience of mental illness.	Findings highlight that stigma and insufficient preparation compromise the quality of emergency care. Targeted training, standardized protocols, adequate resources, and specialist support are essential to reduce stigma and ensure dignified, effective care.
Baminiwatta A, Alahakoon H, Herath NC, Kodithuwakku KM, Nanayakkara T. (2024) [41]	Trait mindfulness, compassion, and stigma towards patients with mental illness: A study among nurses in Sri Lanka.	Quantitative study AQ-9, FFMQ, SCBCS.	405 nurses, Sri Lanka	Higher trait mindfulness was linked to greater willingness to help and less support for avoidance or segregation of people with mental illness. Compassion was inversely related to avoidance and anger, and positively related to pity, helping, and coercion. Mediation analyses showed that compassion partially explained the effects of mindfulness facets (describing, non-reactivity, observing) on stigma-related attitudes.	Trait mindfulness among nurses appears to have a direct buffering effect against several domains of stigma towards psychiatric patients and significant indirect effects through compassion, albeit with small effect sizes.
Alyousef SM, Alhamidi SA. (2023) [42]	Nurse views of obstacles encountered by nurses in Saudi Arabia during the provision of psychiatric care.	Qualitative study/semi-structured interviews and focus group.	10 mental health nurses, Saudi Arabia	Nurses reported multiple obstacles in psychiatric care, including unclear institutional policies, lack of role clarity, low professional confidence, insufficient support, unsafe and stressful work environments, and widespread stigma toward mental health nursing. Stigmatization was experienced not only from patients and families but also from colleagues, the media, and society, reinforcing a negative image of the profession.	Barriers undermine care quality; reducing stigma, strengthening organizational support, and promoting education are essential to advance psychiatric nursing.
Moremi et al. (2024) [43]	Attitudes of primary healthcare nurses towards people living with mental illness in Botswana	Quantitative study Cross-sectional; MICA-4; MAKS	202 primary healthcare nurses, Botswana	51.5% had negative attitudes toward people with mental illness; negative attitudes were associated with being a general (non-specialised) nurse, personal history of mental illness, and lower stigma-related mental health knowledge	Targeted mental health training and anti-stigma programmes are needed to improve nurses’ knowledge and reduce negative attitudes in primary care
Wang et al. (2025) [44]	Exploring the interplay of mental health knowledge, stigma, and social distance among clinical nurses: a study in Liaoning, China	Quantitative study Cross-sectional; SASMIN; Social Distance Scale; Mental Health and Mental Health Knowledge Questionnaire; mediation analysis	628 clinical nurses from five hospitals, China	Nurses showed moderate stigma; 45.5% presented moderate–severe stigma; higher mental health knowledge was associated with lower stigma and reduced social distance, and stigma significantly mediated the relationship between knowledge and social distance	Mental health literacy plays a central role in reducing stigma and social distance; multicomponent educational and stigma-reduction interventions are required in clinical nursing
Jong et al. (2025) [45]	Exploring nurses’ experiences in caring for medical-psychiatric comorbid patients: a qualitative interview study	Qualitative; semi-structured interviews; inductive thematic analysis	16 nurses from internal medicine, surgical and combined wards, university medical centre, Netherlands	Nurses described emotional strain, contextual constraints (time pressure, environment) and a need for competence-building when caring for patients with medical–psychiatric conditions	Organisational support, targeted education and accessible psychiatric consultation are needed to strengthen nurses’ confidence and quality of care for comorbid patients
Mensah (2024) [46]	Perspectives of psychiatric nurses on the stigmatization of mental healthcare in Ghana: a qualitative study	Qualitative; semi-structured interviews; thematic analysis	14 psychiatric nurses, Ghana	Nurses reported social stigma from the public and other professionals, and structural stigma linked to governmental neglect and resource shortages, which devalue psychiatric nursing and hinder care	Associative and structural stigma toward psychiatric nurses and services undermine recruitment, retention and service quality; nurses’ perspectives should inform mental health policy and resource allocation
Hamed et al. (2025) [47]	Stigmatizing attitudes and predictors of empathy toward mentally ill patients among psychiatric and mental health nurses	Quantitative study descriptive correlational cross-sectional; OMS-HC; Perth Empathy Scale; regression analyses	122 psychiatric and mental health nurses, Egypt	70.5% had low stigma (more positive attitudes) and 29.5% high stigma; 64.8% had moderate empathy; higher education and more years of experience predicted both higher empathy and more stigmatizing attitudes; stigma and empathy were negatively correlated	Education, experience and empathy are key correlates of attitudes but do not eliminate stigma; structured anti-stigma and empathy-enhancement programmes are needed even in specialist psychiatric settings

## Data Availability

No new data were created.

## Supplementary Materials

The following supporting information can be downloaded at: https://www.mdpi.com/article/10.3390/nursrep16020050/s1, Table S1. JBI Critical Appraisal of Analytical Cross-Sectional Studies Included in the Review. Table S2. JBI Critical Appraisal of the Quasi-Experimental Study Included in the Review. Table S3. JBI Critical Appraisal of Qualitative Studies Included in the Review. Table S4. JBI Critical Appraisal of the Mixed-Methods Study Included in the Review. Quantitative Component (JBI Cross-Sectional Study Checklist). Table S5. JBI Critical Appraisal of the Mixed-Methods Study Included in the Review. Qualitative Component (JBI Qualitative Checklist). Refs. [57,58,59] are cited in Supplementary Materials.

## Public Involvement Statement

No public involvement was included in any aspect of this research.

## Guidelines and Standards Statement

This manuscript was prepared following methodological elements of the Preferred Reporting Items for Systematic Reviews and Meta-Analyses (PRISMA 2020 statement). The review protocol has been registered and is available at [https://osf.io/9mnjc] (accessed on 24 November 2025).
