# Supplementary material for: Stigma Among Nurses Toward Individuals with Mental Health Conditions: An Integrative Review of Qualitative and Quantitative Studies
Source: Nurs Rep. 2026 Jan 31;16(2):50. doi: 10.3390/nursrep16020050 (PMC12943497; doi:10.3390/nursrep16020050)
Supplement: Supplementary file 1 [file nursrep-16-00050-s001.zip › nursrep-4037462-supplementary.pdf]

Stigma among nurses toward individuals with mental health conditions: a systematized review: PRISMA 2020 Checklist

Section and Topic	Item #	Checklist item	Location where item is reported
**TITLE**			
Title	1	Identify the report as a systematic review.	Title page ("A Systematized Review")
**ABSTRACT**			
Abstract	2	See the PRISMA 2020 for Abstracts checklist.	Abstract (structured format with Background, Methods, Results, Conclusions)
**INTRODUCTION**			
Rationale	3	Describe the rationale for the review in the context of existing knowledge.	Introduction, paragraphs 1–3
Objectives	4	Provide an explicit statement of the objective(s) or question(s) the review addresses.	Final paragraph of Introduction
**METHODS**			
Eligibility criteria	5	Specify the inclusion and exclusion criteria for the review and how studies were grouped for the syntheses.	Section 2.1. Eligibility Criteria
Information sources	6	Specify all databases, registers, websites, organisations, reference lists and other sources searched or consulted to identify studies. Specify the date when each source was last searched or consulted.	Section 2.2. Search Strategy (databases and search dates)
Search strategy	7	Present the full search strategies for all databases, registers and websites, including any filters and limits used.	Section 2.2. Search Strategy (keywords, boolean terms)
Selection process	8	Specify the methods used to decide whether a study met the inclusion criteria of the review, including how many reviewers screened each record and each report retrieved, whether they worked independently, and if applicable, details of automation tools used in the process.	Section 2.3. Study Selection
Data collection process	9	Specify the methods used to collect data from reports, including how many reviewers collected data from each report, whether they worked independently, any processes for obtaining or confirming data from study investigators, and if applicable, details of automation tools used in the process.	Section 2.4. Data Extraction
Data items	10a	List and define all outcomes for which data were sought. Specify whether all results that were compatible with each outcome domain in each study were sought (e.g. for all measures, time points, analyses), and if not, the methods used to decide which results to collect.	Section 2.4. Data Extraction; Table 2 (variables extracted)
	10b	List and define all other variables for which data were sought (e.g. participant and intervention characteristics, funding sources). Describe any assumptions made about any missing or unclear information.	Section 2.4. Data Extraction; Table 2
Study risk of bias assessment	11	Specify the methods used to assess risk of bias in the included studies, including details of the tool(s) used, how many reviewers assessed each study and whether they worked independently, and if applicable, details of automation tools used in the process.	Section 2.5. Quality Appraisal (JBI tools used; two reviewers)
Effect measures	12	Specify for each outcome the effect measure(s) (e.g. risk ratio, mean difference) used in the synthesis or presentation of results.	Not applicable (narrative synthesis; no effect measures

Section and Topic	Item #	Checklist item	Location where item is reported
			used)
Synthesis methods	13a	Describe the processes used to decide which studies were eligible for each synthesis (e.g. tabulating the study intervention characteristics and comparing against the planned groups for each synthesis (item #5)).	Section 2.6. Data Synthesis (quanti vs. quali segmentation)
	13b	Describe any methods required to prepare the data for presentation or synthesis, such as handling of missing summary statistics, or data conversions.	Section 2.6. Data Synthesis (narrative synthesis, no data transformation)
	13c	Describe any methods used to tabulate or visually display results of individual studies and syntheses.	Table 2; Supplementary Materials (JBI appraisal tables)
	13d	Describe any methods used to synthesize results and provide a rationale for the choice(s). If meta-analysis was performed, describe the model(s), method(s) to identify the presence and extent of statistical heterogeneity, and software package(s) used.	Section 2.6. Data Synthesis (narrative approach; no meta-analysis)
	13e	Describe any methods used to explore possible causes of heterogeneity among study results (e.g. subgroup analysis, meta-regression).	Not applicable (no statistical heterogeneity assessment)
	13f	Describe any sensitivity analyses conducted to assess robustness of the synthesized results.	Not applicable (no meta-analysis conducted)
Reporting bias assessment	14	Describe any methods used to assess risk of bias due to missing results in a synthesis (arising from reporting biases).	Not applicable (no quantitative synthesis performed)
Certainty assessment	15	Describe any methods used to assess certainty (or confidence) in the body of evidence for an outcome.	Results → Section "Quality Appraisal of Included Studies" (moderate overall quality)
**RESULTS**			
Study selection	16a	Describe the results of the search and selection process, from the number of records identified in the search to the number of studies included in the review, ideally using a flow diagram.	Section 3.1. General Characteristics of the Included Studies; Figure 1 (PRISMA flow)
	16b	Cite studies that might appear to meet the inclusion criteria, but which were excluded, and explain why they were excluded.	Section 3.1. General Characteristics of the Included Studies (20 excluded for not meeting criteria)

Section and Topic	Item #	Checklist item	Location where item is reported
Study characteristics	17	Cite each included study and present its characteristics.	Table 2; Section 3.2 (study summaries)
Risk of bias in studies	18	Present assessments of risk of bias for each included study.	Results → “Quality Appraisal of Included Studies”; Supplementary Materials (JBI tables)
Results of individual studies	19	For all outcomes, present, for each study: (a) summary statistics for each group (where appropriate) and (b) an effect estimate and its precision (e.g. confidence/credible interval), ideally using structured tables or plots.	Sections 3.3 (Quantitative Findings) and 3.4 (Qualitative Findings); Table 2
Results of syntheses	20a	For each synthesis, briefly summarise the characteristics and risk of bias among contributing studies.	Section 3.5. Synthesis of Findings; “Quality Appraisal of Included Studies”
	20b	Present results of all statistical syntheses conducted. If meta-analysis was done, present for each the summary estimate and its precision (e.g. confidence/credible interval) and measures of statistical heterogeneity. If comparing groups, describe the direction of the effect.	Not applicable (no meta-analysis)
	20c	Present results of all investigations of possible causes of heterogeneity among study results.	Not applicable
	20d	Present results of all sensitivity analyses conducted to assess the robustness of the synthesized results.	Not applicable
Reporting biases	21	Present assessments of risk of bias due to missing results (arising from reporting biases) for each synthesis assessed.	Not applicable
Certainty of evidence	22	Present assessments of certainty (or confidence) in the body of evidence for each outcome assessed.	Results → “Quality Appraisal of Included Studies”; Discussion (consideration of moderate evidence)
**DISCUSSION**			
Discussion	23a	Provide a general interpretation of the results in the context of other evidence.	Section 4. Discussion, paragraphs 1–3
	23b	Discuss any limitations of the evidence included in the review.	Section 4. Discussion (paragraph describing methodological variability and study quality)
	23c	Discuss any limitations of the review processes used.	Section 4.

Section and Topic	Item #	Checklist item	Location where item is reported
			Discussion (limitations related to search, availability of full texts)
	23d	Discuss implications of the results for practice, policy, and future research.	Section 4. Discussion; Section 5. Conclusions
**OTHER INFORMATION**			
Registration and protocol	24a	Provide registration information for the review, including register name and registration number, or state that the review was not registered.	Section 2 registered on OSF (https://osf.io/9mnjc).
	24b	Indicate where the review protocol can be accessed, or state that a protocol was not prepared.	Section 2 registered on OSF (https://osf.io/9mnjc).
	24c	Describe and explain any amendments to information provided at registration or in the protocol.	No amendments were made to the registered protocol.
Support	25	Describe sources of financial or non-financial support for the review, and the role of the funders or sponsors in the review.	Funding statement at end of manuscript
Competing interests	26	Declare any competing interests of review authors.	Conflict of Interest statement at end of manuscript
Availability of data, code and other materials	27	Report which of the following are publicly available and where they can be found: template data collection forms; data extracted from included studies; data used for all analyses; analytic code; any other materials used in the review.	Data Availability Statement ("All data extracted from published studies; JBI appraisal tables available in Supplementary Materials")

From: Page MJ, McKenzie JE, Bossuyt PM, Boutron I, Hoffmann TC, Mulrow CD, et al. The PRISMA 2020 statement: an updated guideline for reporting systematic reviews. BMJ 2021;372:n71. doi: 10.1136/bmj.n71.
This work is licensed under CC BY 4.0. To view a copy of this license, visit https://creativecommons.org/licenses/by/4.0/

Stigma among nurses toward individuals with mental health conditions: a systematized review.

Supplementary material.

Ruth Auxiliadora Díaz-Melián, Jesús Manuel Quintero-Febles and Alfonso Miguel García-Hernández

Table S1. JBI Critical Appraisal of Analytical Cross-Sectional Studies Included in the Review

Author (Year)	Title	Item 1	Item 2	Item 3	Item 4	Item 5	Item 6	Item 7	Item 8	Quality
Halter MJ. (2008) [12]	Perceived characteristics of psychiatric nurses: stigma by association.	Yes	Unclear	Yes	Yes	Unclear	No	Yes	Yes	Moderate 5/8
McIntosh JT. (2023) [14]	Emergency department nurses' perceptions of caring behaviors toward individuals with mental illness: A secondary analysis.	Yes	Yes	Yes	Yes	Yes	Yes	Yes	Yes	High 8/8
McIntosh JT. (2023) [15]	Illuminating Emergency Nurses' Perceptions of Stigma, Attribution, and Caring Behaviors Toward People with Mental Illness Through the Lens of Individualized Care: A Cross-sectional Study.	Yes	Yes	Yes	Yes	Yes	Yes	Yes	Yes	High 8/8
Björkman T, Angelman T, Jönsson M. (2008) [16]	Attitudes towards people with mental illness: a cross-sectional study among nursing staff in psychiatric and somatic care.	Yes	Unclear	Yes	Yes	Unclear	No	Yes	Yes	Moderate 5/8
Hamdan-Mansour AM, Wardam LA. (2009) [17]	Attitudes of Jordanian mental health nurses toward mental illness and patients with mental illness.	Yes	Unclear	Yes	Yes	Unclear	Unclear	Yes	Yes	Moderate 5/8
Chambers M, Guise V, Välimäki M, Botelho MA, Scott A, Staniulienė V, et al. (2010) [18]	Nurses' attitudes to mental illness: a comparison of a sample of nurses from five European countries.	Yes	Yes	Yes	Yes	Yes	Yes	Yes	Yes	High 8/8
Ihalainen-Tamlander N, Vähäniemi A, Löyttyniemi E, Suominen T, Välimäki M. (2016) [19]	Stigmatizing attitudes in nurses towards people with mental illness: a cross-sectional study in primary settings in Finland.	Yes	Unclear	Yes	Yes	Unclear	No	Yes	Yes	Moderate 5/8
Fontesse S, Rimez X, Maurage P. (2021) [21]	Stigmatization and dehumanization perceptions towards psychiatric patients	Yes	Yes	Yes	Yes	Yes	Unclear	Yes	Yes	High 7/8

	among nurses: A path-analysis approach.									
Ben Natan M, Drori T, Hochman O. (2015) [22]	Associative Stigma Related to Psychiatric Nursing Within the Nursing Profession.	Yes	Unclear	Yes	Yes	Unclear	No	Yes	Yes	Moderate 5/8
Vedaste B, Smith A A H. (2016) [23]	In principle, yes, in application, no': Rwandan nurses' support for integration of mental health services.	Yes	Unclear	Yes	Yes	Unclear	No	Yes	Yes	Moderate 5/8
Al-Awadhi A, Atawneh F, Alalyan MZY, Shahid AA, Al-Alkhadhari S, Zahid MA. (2017) [24]	Nurses' attitude towards patients with mental illness in a general hospital in Kuwait.	Yes	Unclear	Yes	Yes	Unclear	No	Yes	Yes	Moderate 5/8
Ebrahimi H, Jafarabadi MA, Areshtanab HN, Pourabbas M, Dehghan A, Vahidi M. (2017) [26]	Comparing mental illness stigma among nurses in psychiatric and non-psychiatric wards in Tabriz University of medical sciences.	No	Unclear	Yes	Yes	No	No	Yes	Unclear	Low 3/8
Weare R, Green C, Olasoji M, Plummer V. (2019) [29]	ICU nurses feel unprepared to care for patients with mental illness: A survey of nurses' attitudes, knowledge, and skills.	Yes	Yes	Yes	Yes	Yes	Yes	Yes	Yes	High 8/8
Sahile Y, Yitayih S, Yeshanew B, Ayelegne D, Mihiretu A. (2019) [31]	Primary health care nurses' attitude towards people with severe mental disorders in Addis Ababa, Ethiopia: A cross-sectional study.	No	Unclear	Yes	Yes	No	No	Yes	Unclear	Low 3/8
Shahif S, Idris DR, Lupat A, Abdul Rahman H. (2019) [32]	Knowledge and attitude towards mental illness among primary healthcare nurses in Brunei: A cross-sectional study.	Yes	Unclear	Yes	Yes	Unclear	No	Yes	Yes	Moderate 5/8
Grover S, Sharma N, Mehra A. Stigma for Mental Disorders among Nursing Staff in a Tertiary Care Hospital. (2020) [33]	Stigma for Mental Disorders among Nursing Staff in a Tertiary Care Hospital.	Yes	Yes	Yes	Yes	Yes	Yes	Yes	Yes	High 8/8
Román-Sánchez D, Paramio-Cuevas JC, Paloma-Castro O, Palazón-Fernández JL, Lepiani-Díaz I, de la Fuente Rodríguez JM, et al. (2022) [35]	Empathy, Burnout, and Attitudes towards Mental Illness among Spanish Mental Health Nurses.	Yes	Yes	Yes	Yes	Yes	Yes	Yes	Yes	High 8/8
Baminiwatta A, Alahakoon H, Herath NC,	Trait mindfulness, compassion, and stigma towards	Yes	Unclear	Yes	Yes	Unclear	No	Yes	Yes	Moderate 5/8

Kodithuwakku KM, Nanayakkara T. (2024) [37]	patients with mental illness: A study among nurses in Sri Lanka.									
Moremi et al. (2024)	Attitudes of primary healthcare nurses towards people living with mental illness in Botswana	Yes	Yes	Yes	Yes	Yes	Yes	Yes	Yes	High 8/8
	Exploring the interplay of mental health knowledge, stigma, and social distance among clinical nurses: a study in Liaoning, China	Yes	Yes	Yes	Yes	Yes	No	Yes	Yes	Moderate 7/8
	Stigmatizing attitudes and predictors of empathy toward mentally ill patients among psychiatric and mental health nurses	Yes	Yes	Yes	Yes	Yes	Yes	Yes	Yes	High 8/8

- Item 1: Inclusion/exclusion criteria clearly defined
- Item 2: Participants and setting described in detail
- Item 3: Exposure measured validly and reliably
- Item 4: Objective criteria used for outcome measurement
- Item 5: Confounding factors identified
- Item 6: Strategies to address confounding
- Item 7: Outcomes measured validly and reliably
- Item 8: Statistical analysis appropriate

Bibliography

Moola S, Munn Z, Tufanaru C, Aromataris E, Sears K, Sfetcu R, Currie M, Qureshi R, Mattis P, Lisy K, Mu P-F. Chapter 7: Systematic reviews of etiology and risk . In: Aromataris E, Munn Z (Editors). JBI Manual for Evidence Synthesis. JBI, 2020. Available from https://synthesismanual.jbi.global

Table S2. JBI Critical Appraisal of the Quasi-Experimental Study Included in the Review

Author (Year)	Title	1	2	3	4	5	6	7	8	9	Quality
Ng YP, Rashid A, O'Brien F. (2017) [25]	Determining the effectiveness of a video-based contact intervention in improving attitudes of Penang primary care nurses towards people with mental illness.	Yes	Yes	No	Yes	Unclear	Yes	Yes	Yes	Unclear	Moderate 6/9

Legend:

- Item 1: Clear inclusion/exclusion criteria
- Item 2: Participants and setting clearly described
- Item 3: Randomization or appropriate assignment
- Item 4: Baseline characteristics reported
- Item 5: Confounding factors identified and controlled
- Item 6: Valid and reliable outcome measures
- Item 7: Follow up and completion rates adequate
- Item 8: Appropriate statistical analysis
- Item 9: Analysis by intention to treat

Bibliography

Barker TH, Habibi N, Aromataris E, Stone JC, Leonardi-Bee J, Sears K, et al. The revised JBI critical appraisal tool for the assessment of risk of bias quasi-experimental studies. JBI Evid Synth. 2024;22(3):378-88. Available from: https://jbi.global/sites/default/files/2024-04/2_JBI%20checklist%20for%20quasi-experimental%20studies.docx

Table S3. JBI Critical Appraisal of Qualitative Studies Included in the Review

Author (Year)	Title	1	2	3	4	5	6	7	8	9	10	Quality
Zolnierek CD, Clingerman EM. (2012) [13]	A medical–surgical nurse's perceptions of caring for a person with severe mental illness.	U	Y	Y	Y	Y	N	N	Y	Y	Y	7/10 Moderate quality
Sercu C, Ayala RA, Bracke P. (2015) [20]	How does stigma influence mental health nursing identities? An ethnographic study of the meaning of stigma for nursing role identities in two Belgian Psychiatric Hospitals.	Y	Y	Y	Y	Y	U	U	Y	Y	Y	8/10 High quality
Harrison CA, Hauck Y, Ashby R. (2017) [27]	Breaking down the stigma of mental health nursing: A qualitative study reflecting opinions from Western Australian nurses.	U	Y	Y	Y	Y	U	U	Y	Y	Y	7/10 Moderate quality
Brunero S, Buus N, West S. (2017) [28]	Categorising Patients Mental Illness by Medical Surgical Nurses in the General Hospital Ward: A Focus Group Study.	Y	Y	Y	Y	Y	U	U	Y	Y	Y	8/10 High quality
Mendenhall E, Isaiah G, Nelson B, Musau A, Koon AD, Smith L, et al. (2018) [30]	Nurses' perceptions of mental healthcare in primary-care settings in Kenya.	U	Y	Y	Y	Y	U	N	Y	Y	Y	7/10 Moderate quality
García-Carpintero Blas E, Gómez-Moreno C, Moreno-Gomez-Toledano R, Ayuso-Del-Olmo H, Rodrigo-Guijarro E, Polo-Martínez S, et al. (2023) [36]	Help! Caring for People with Mental Health Problems in the Emergency Department: A Qualitative Study.	Y	Y	Y	Y	Y	U	U	Y	Y	Y	8/10 High quality

Alyousef SM, Alhamidi SA. (2023) [38]	Nurse views of obstacles encountered by nurses in Saudi Arabia during the provision of psychiatric care.	U	Y	Y	Y	Y	U	U	Y	Y	Y	7/10 Moderate quality
Jong et al. (2025)	Exploring nurses' experiences in caring for medical psychiatric comorbid patients: a qualitative interview study	Y	Y	Y	Y	Y	Y	Y	Y	Y	Y	10/10 High quality
Mensah (2024)	Perspectives of psychiatric nurses on the stigmatization of mental healthcare in Ghana: a qualitative study	U	Y	Y	Y	Y	Y	U	Y	Y	Y	8/10 High quality

Legend:

Y = Yes

U = Unclear

N = No

- Item 1: Congruity between the stated philosophical perspective and the research methodology
- Item 2: Congruity between the research methodology and the research question or objectives
- Item 3: Congruity between the research methodology and the methods used to collect data
- Item 4: Congruity between the research methodology and the representation and analysis of data
- Item 5: There is congruence between the research methodology and the interpretation of results
- Item 6: Locating the researcher culturally or theoretically
- Item 7: Influence of the researcher on the research, and vice-versa, is addressed
- Item 8: Representation of participants and their voices
- Item 9: Ethical approval by an appropriate body
- Item 10: Relationship of conclusions to analysis, or interpretation of the data

Bibliography

Lockwood C, Munn Z, Porritt K. Qualitative research synthesis: methodological guidance for systematic reviewers utilizing meta-aggregation. Int J Evid Based Healthc. 2015;13(3):179–187 Available from: https://jbi.global/sites/default/files/2021-10/Checklist_for_Qualitative_Research.docx

Table S4. JBI Critical Appraisal of the Mixed-Methods Study Included in the Review. Quantitative Component (JBI Cross-Sectional Study Checklist)

Author (Year)	Title	Item 1	Item 2	Item 3	Item 4	Item 5	Item 6	Item 7	Item 8	Quality
Waddell C, Graham JM, Pachkowski K, Friesen H. (2020) [34]	Battling Associative Stigma in Psychiatric Nursing	U	Y	U	U	N	N	U	Y	Low 2/8

Table S5. JBI Critical Appraisal of the Mixed-Methods Study Included in the Review. Qualitative Component (JBI Qualitative Checklist)

Author (Year)	Title	1	2	3	4	5	6	7	8	9	10	Quality
Waddell C, Graham JM, Pachkowski K, Friesen H. (2020) [34]	Battling Associative Stigma in Psychiatric Nursing	U	Y	Y	Y	Y	N	N	Y	Y	Y	7/10 Moderate

Y = Yes

U = Unclear

N = No
